# E-Learning amid the COVID-19 Lockdown: Standpoint of Medical and Dental Undergraduates

**DOI:** 10.12669/pjms.37.1.3124

**Published:** 2021

**Authors:** Aiza Anwar, Hajra Mansoor, Danyal Faisal, Huma Saeed Khan

**Affiliations:** 1 Aiza Anwar, Medical Student 2^nd^ Year MBBS. CMH Lahore Medical College & Institute of Dentistry, Lahore, Pakistan; 2 Hajra Mansoor, Medical Student 2^nd^ Year MBBS. CMH Lahore Medical College & Institute of Dentistry, Lahore, Pakistan; 3 Danyal Faisal, Medical Student 2^nd^ Year MBBS. CMH Lahore Medical College & Institute of Dentistry, Lahore, Pakistan; 4 Dr. Huma Saeed Khan, M.Phil., Associate Professor, Department of Physiology, CMH Lahore Medical College & Institute of Dentistry, Lahore, Pakistan

**Keywords:** COVID-19, E-learning, Medical education, Online learning, Readiness for E-learning

## Abstract

**Background and Objective:**

The corona-virus (COVID-19) pandemic has had devastating impacts on not only the health and socio-economic conditions but also on the education sector in various countries. Consequently, the world of education entered a new era of E-learning. This descriptive study, thus, aims to evaluate the standpoint of medical and dental undergraduates regarding E-learning amid the Covid-19 lockdown.

**Methods:**

A descriptive online questionnaire was used to gather responses via Google forms. The questionnaire consisted of questions categorized as technology access, online skills and relationships, students’ views, and student’s perception of possible advantages of E-learning. Two hundred and eighty three medical and dental students of CMH Lahore Medical College participated in the study.

**Results:**

One hundred and seventy nine female and 104 male students responded to the questionnaire. The results of the study showed students encouraging the accommodation of E-learning into current teaching practices. Students agreed online study material not only provides flexibility in the learning process (*p*=0.003) but also saves time (*p* = 0.012). Female participants showed a more positive readiness response towards e learning than males (*p* =0.001). Results also showed students have easier access to online teaching resources. Regarding online lectures, the students believe that online lectures were more organized (*p*= 0.001) and stimulated student interest (*p*=0.026). Students believe that frequent participation in the learning process is important for success in online education (*p*=0.002).

**Conclusion:**

Undergraduate medical and dental students from the said private medical college are well equipped and ready to shift towards online education.

## INTRODUCTION

COVID-19, beginning in Wuhan, China, in December 2019 remains an imperative problem affecting millions worldwide.^1^ It was declared a pandemic on March 11, 2020 by the World Health Organization (WHO) and by March 25, 2020 the total confirmed cases that were reported rose to 414,179.^2^ Together with infecting individuals’ health, COVID-19 continues to damage the socio-economic conditions and education of any country it has touched, thus being more than just a medical emergency.^1^,^3^,^4^

Countries lost no time in trying to combat the adversity by imposing strict restrictions on large gatherings, instructing individuals to practise social distancing, launching health promotion and disease prevention programs and so forth.^4^ Flights to and from China were suspended by most countries and the governments urged on banning travel until the situation alleviates.^2^,^5^

With limitations on gatherings came the closure of malls, restaurants, workplaces, and educational institutions. Shutdown of educational institutions has caused the world to move towards E-learning.^6^,^7^ This, in turn, poses another challenge for countries with a low socio-economic status, like Pakistan.^8^

Pakistan, as it continues to face a rise in the number of COVID-19 cases, had its institutions switch to E-learning as per instructions by the Higher Education Commission (HEC) of the country.^9^ Under this list of institutions come the medical colleges of the country with both, the medical undergraduates and the lecturers, being affected by the shift from ‘on-campus’ classes to virtual lectures.^10^,^11^ Not being very familiar with such teaching methodologies, instructors and students struggle to consolidate these with their plan of continuing medical education while maintaining the quality of it.^12^,^13^

Lack of expertise in operating the electronic resources and the concomitant problem of limited access to the internet, computers and the other facilities due to social and economic setbacks is an obstacle for online learning and teaching.^14^ Hence, this research was conducted with an aim to obtain the standpoint of medical and dental students of a private medical college in Pakistan regarding the transition to online learning, its effectiveness and their satisfaction over what has been achieved since the shift. This will add to the current studies concerning the readiness of students for adapting to this new mode of education. It is an especially important inclusion to literature that is aimed towards the incorporation of online learning in medical education.

## METHODS

This descriptive cross sectional research was conducted amongst the undergraduate students of CMH-Lahore Medical College and Institute of Dentistry. With undergraduate students from both MBBS and BDS taking part in this, the research was conducted from May to June 2020. A sample size of 283 participants was used which was calculated from Rao soft using the formula Z2p (1 – p) / m2 and a confidence level of 95%. The tolerable margin of error was kept at 5%. A 20 item questionnaire was used to gather responses via Google forms. Informed consent was taken from all participants prior to data collection. The response settings were done to restrict only one response from a person. The questionnaire contained statements used to assess the stand point of students regarding E-Learning. The statements were categorized into categories as follows: technology access, online skills and relationships, students’ view and student’s perceptions of possible advantages of E-Learning. With each statement, a 5 point Likert scale response was used from strongly agree (SA) to strongly disagree (SD).^15^ The original version of the questionnaire used was developed by Watkins et al.^15^ Permission to use questionnaire was obtained from the author through email. Ethical approval for this research was obtained from ethical review committee of CMH Lahore Medical College and Institute of Dentistry, Lahore (Reference Number 57/ERC/CMHLMC Dated 24th May 2020). All data gathered was entered into and analyzed by SPSS version 25. Frequencies and percentages were obtained for the responses and Chi-square test was used to compare responses based on gender. A p-value of < 0.05 was considered statistically significant.

## RESULTS

Out of the 283 responses received, 179 were female students while 104 were male. The questions, each with options from strongly disagree to strongly agree, were divided into the following categories for presenting results: Technology access with online skills and relationships; Students point of view on online lectures; and Students’ perception of the possible advantage of E-learning.

Regarding technology access, most participants showed to have access to technology and also possessed the basic skills of operating it. Percentage of participants with access to technological equipment was high (strongly agree=39.2% and agree=39.9%). Response of participants about possessing online skills and relationships was encouraging (44.9% answered strongly agree and 38.9% answered agree). The difference between the viewpoint of male students and female students in this category of variables was statistically different for each response as shown in Table-I.

**Table-I:** Technology access with online skills and relationships (n=283).

	Gender	SA	A	N	D	SD	p-value
I have access to a mobile/laptop/computer with internet access	Male f(%)	26 9.2%	51 18%	4 1.4%	13 4.6%	10 3.5%	0.004
	Female f(%)	85 30%	62 22%	7 2.5%	17 6%	8 2.8%	
I have the basic skills to operate a mobile/ laptop/ computer (saving files, creating folders)	Male f (%)	29 10%	49 17%	4 1.4%	15 5.3%	7 2.5%	0.001
	Female f (%)	98 35%	61 22%	5 1.8%	9 3.2%	6 2.1%	
I have the basic skills for finding my way around the internet e.g., using search engines etc.	Male f(%)	32 11%	42 15%	7 2.5%	14 4.9%	9 3.2%	0.001
	Female f (%)	92 33%	65 23%	6 2.1%	14 4.9%	2 0.7%	
I can send an email with a file attached	Male f(%)	37 13%	43 15%	8 2.8%	9 3.2%	7 2.5%	0.001
	Female f (%)	103 36%	60 21%	4 1.4%	9 3.2%	3 1.1%	
I think that I would be able to communicate effectively with others using online technologies	Male f (%)	17 6%	33 12%	20 7.1%	11 3.9%	23 8.1%	0.001
	Female f (%)	66 23%	61 22%	23 8.1%	17 6%	12 4.2%	
I think that I would be able to use online tools to work on assignments	Male f (%)	15 5.3%	35 12%	21 7.4%	13 4.6%	20 7.1%	0.001
	Female f (%)	63 22%	65 23%	22 7.8%	18 6.4%	11 3.9%	
I think that I would be able to provide timely responses to teachers	Male f (%)	12 4.2%	31 11%	21 7.4%	13 4.6%	27 9.5%	0.001
	Female f(%)	52 18%	61 22%	32 11%	23 8.1%	11 3.9%	
I think that I would be able to ask/answer questions	Male f(%)	16 5.7%	22 7.8%	23 8.1%	20 7.1%	23 8.1%	0.001
	Female f(%)	50 18%	58 20%	30 11%	28 9.9%	13 4.6%	

SA= Strongly agree; A= Agree; N= Neutral; D= Disagree; SD= Strongly disagree.p -value for mean responses between males and females by Chi-square test.p -value< 0.05 considered statistically significant.

The next category, that is, students’ point of view on online lectures, showed a positive response with 43.1% of participants agreeing that instructors were available and helpful. Along with this, 37.5% of the individuals could understand course related information via online videos. Furthermore, it was shown that 39.2% of the participants agreed on presentations being clear but 30.0% of these were not sure about whether the instructor stimulated interest and 28.6% of them disagreed. Further analysis of the statistics presented evidence of 32.2% of participants agreeing over frequent participation being pivotal for success in online lectures with 23.7% remaining unsure. The difference between the viewpoint on male students and female students is shown in Table-II.

**Table-II:** Students point of view on online lectures (n=283).

	Gender	SA	A	N	D	SD	p-value
Presentations were clear and organized	Male f(%)	4 1.4%	34 12%	22 7.8%	25 8.8%	19 6.7%	0.001
	Female f(%)	23 8.1%	77 27%	34 12%	36 13%	9 3.2%	
Instructor stimulated student interest	Male f(%)	1 0.4%	17 6%	34 12%	28 9.9%	24 8.5%	0.026
	Female f(%)	11 3.9%	42 15%	51 18%	53 19%	22 7.8%	
Instructor was available and helpful	Male f(%)	7 2.5%	38 13%	28 9.9%	20 7.1%	11 3.9%	0.346
	Female f (%)	16 5.7%	84 30%	36 13%	30 11%	13 4.6%	
I think that I would be able to understand/deliver course related information when it is presented in video format rather than online lecture	Male f(%)	19 6.7%	38 13%	19 6.7%	15 5.3%	13 4.6%	0.199
	Female f(%)	51 18%	68 24%	22 7.8%	24 8.5%	14 4.9%	
Frequent participation throughout the learning process is important to my success in online lectures	Male f(%)	7 2.5%	27 9.5%	26 9.2%	23 8.1%	21 7.4%	0.002
	Female f(%)	29 10%	64 23%	41 14%	32 11%	13 4.6%	

SA= Strongly agree; A= Agree; N= Neutral; D= Disagree; SD= Strongly disagree.p-value for mean responses between males and females by Chi-square test.p-value< 0.05 considered statistically significant.

The final category, involving both students’ perception of possible advantages of E-learning, indicated that 38.2% individuals agreed about E-learning providing flexibility in learning, however, a mixed response was observed regarding better understanding of lectures through online classes as statistics reveal 30.0% agreeing while 27,6% disagreeing. Additionally, a higher percentage (33.2) of participants disagreed over the possible benefit that E-learning can improve communication between teachers and students. A higher percentage of students strongly agreed (17.7%) and agreed (31.4%) on the possible advantage of saving time. The comparison of these responses between males and females showed a statistically significant difference as shown in Table-III.

**Table-III:** Students’ perception of the possible advantage of E-learning (n=283).

	Gender	SA	A	N	D	SD	p-value
Provides flexibility in the learning process	Male f (%)	11 3.9%	32 11%	18 6.4%	26 9.2%	17 6%	0.003
	Female f (%)	29 10%	76 27%	29 10%	38 13%	7 2.5%	
Provides students with a better understanding of the lessons	Male f(%)	8 2.8%	22 7.8%	21 7.4%	30 11%	23 8.1%	0.006
	Female f(%)	19 6.7%	63 22%	34 12%	48 17%	15 5.3%	
Improves communication	Male f (%)	8 2.8%	22 7.8%	18 6.4%	34 12%	22 7.8%	0.130
	Female f(%)	18 6.4%	47 17%	36 13%	60 21%	18 6.4%	
Saves time by using online resources	Male f(%)	16 5.7%	25 8.8%	20 7.1%	19 6.7%	24 8.5%	0.012
	Female f(%)	34 12%	64 23%	30 11%	35 12%	16 5.7%	

SA= Strongly agree; A= Agree; N= Neutral; D= Disagree; SD= Strongly disagree.p -value for mean responses between males and females by Chi-square test.p-value< 0.05 considered statistically significant.

## DISCUSSION

Our study shows that students believe e-learning has increased their motivation and made them more engaged with the course content which is similar to previous findings by Mukhtar K in the literature.^14^ It was found that students participate more actively in class and show a better performance in assessment of core knowledge.^16^ A study by Abbasi S shows that e-learning is discerned to be less effective as compared to face to face learning, whereas our findings suggest that even with a lack of face to face learning students could provide timely responses to teachers and also ask questions.^17^ However, previously, e-learning was always combined with tasks that had to be performed in person like small group discussions and demonstrations or problem based learning as reported by Burac M.^18^ Students also displayed that online teaching and the greater available of resources provided them with a better understanding of lessons. This suggests that e-learning has helped improve teaching concepts and has led to a reform with a student centred model being preferred by instructors.^19^ The Covid-19 pandemic has led to transition to an online medium of instructions not only for preclinical years, but also in clinical sciences which has deferred the attainment of skills in many cases. According to literature review, there is continuous evolvement of options which may lead to modification of the educational calendar for students in their clinical years or instructors may employ virtual cases for demonstrations.^20^ Based on our findings, this can be possibility as a majority agrees that online teaching stimulates student interest and is an efficient means for understanding as well as communication.

A vast majority of the study participants have responded with encouraging responses about their readiness for online education. This is in stark contrast with the findings presented by Alipio M from a study conducted in Philippines. According to the study, there is much less access to technology, and females are more likely to be deprived of this access especially in the rural areas.^1^ The current study, however, was conducted on a sample population of medical and dental undergraduates from a private medical college, and thus these belong to a comparatively better socioeconomic status. Also, the female participants in the current study have shown a higher number of positive responses than males for access to technology, use of basic computer programs and online search techniques.

In these unprecedented times, there is a need to provide for a more flexible learning environment for online education. The participants of the current study have agreed with the notion that online study material provides flexibility in the learning process, makes understanding easier and also saves time. This is in line with the findings reported by Chick et al. Their study population comprised of surgical residents, and the learning and teaching method used was a flipped class room model.^21^

### Limitations of the study

The rural or urban place of residence was not considered, which may have an impact on the quality of internet service being provided in that area.^22^ The quality assurance methods for these online sessions will also be need to studied separately, as these make a part and parcel for the online sessions.^23^ Online assessments also present a vast area of medical education that has not been explored in this study.

## CONCLUSION

Undergraduate medical and dental students from the said private medical college are fairly ready and equipped to shift towards online education. However, more studies need to be conducted to evaluate the impact of online teaching on assessments. Training of teachers and students in the use of online study platforms also need to be explored. Moreover, the impact of this shift on teacher-student relationships outside of the classroom needs to be assessed to appraise the efficacy of E-learning in the medical field in the long term.

### Authors’ Contribution:

**AA:** Conceived, designed and did statistical analysis & editing of manuscript.

**AA, HM, DF:** Did data collection and manuscript writing.

**HSK:** Did overall supervision, final drafting, revision and final approval of manuscript.
